# The impact of obstructive sleep apnea on cognitive decline in adults with Down Syndrome – preliminary findings

**DOI:** 10.1002/alz.092580

**Published:** 2025-01-09

**Authors:** Ana Paula Assunção Cecilio, Claudia Lopes Carvalho, Aline Souza Gonçalves, Rosa Hasan, Luiz Ubirajara Sennes, Orestes Vicente Forlenza

**Affiliations:** ^1^ Laboratory of Neuroscience (LIM‐27), Department and Institute of Psychiatry, Faculty of Medicine, University of São Paulo, São Paulo, São Paulo Brazil; ^2^ Laboratory of Clinical Neurophysiology, Department and Institute of Psychiatry, Faculty of Medicine, University of São Paulo, São Paulo, São Paulo Brazil; ^3^ Department of Otorhinolaryngology, Faculty of Medicine, University of São Paulo‐Brazil, São Paulo, São Paulo Brazil

## Abstract

**Background:**

*Sleep*‐related *breathing disorders* are commonly reported in the Down Syndrome (DS) population, but data on its prevalence and severity are scarce, especially for the adult population. The increase in life expectancy and premature aging in patients with DS reinforces the need for an assessment of sleep quality. This study evaluated sleep‐disordered breathing in adults with DS using sleep measures by polysomnography. The aim of this study is to define the association between the severity of obstructive sleep apnea (OSA) in adults with DS and the clinical diagnoses of stable cognition *versus* prodromal dementia and dementia. Furthermore, it was possible to correlate sleep respiratory variables in these groups.

**Method:**

All participants underwent a sleep study Type 1 Polysomnography, analyzed according to the American Academy of Sleep Medicine (AASM). The diagnostic criteria for OSA and its severity were based on the description of the Diagnostic and Statistical Manual of Mental Disorders (DSM‐5). A multidisciplinary consensus including psychiatrists and geriatricians classified the participants into three diagnostic groups: stable cognition, prodromal demetia, and dementia. A neuropsychological assessment was carried out, according to the Cambridge Examination for Mental Disorders of Older People with Down’s Syndrome and Others with Intellectual Disabilities (CAMDEX‐DS). The Kruskal‐Wallis test was applied to evaluate our results.

**Result:**

3 participants had dementia (mean AHI 17,80 events/h), 7 were classified as prodromal dementia (mean AHI 42,60 events/h) and 28 were in the stable cognition group (mean AHI 36,42 events/h). It was not possible to determine statistically significant evidence between the AHI and the diagnoses. The correlation between respiratory sleep variables demonstrated a strong association between AHI, AHI in NREM sleep and ODI values. Furthermore, the higher the AHI or ODI values, the lower the minimum oxyhemoglobin saturation. However, it was not possible to observe a correlation with the mean oxyhemoglobin saturation.

**Conclusion:**

Studies show that OSA is the main sleep disorder in the DS population, demonstrating the importance of early diagnosis. In our analysis, it was not possible to define a correlation between OSA and clinical diagnoses, possibly because there are few non‐control cases. Being preliminary data, clinical and polysomnographic follow‐up will support our preliminary hypotheses.

